# Strain echocardiography in septic shock – a comparison with systolic and diastolic function parameters, cardiac biomarkers and outcome

**DOI:** 10.1186/s13054-015-0857-1

**Published:** 2015-03-26

**Authors:** Lina De Geer, Jan Engvall, Anna Oscarsson

**Affiliations:** Department of Intensive Care Medicine, Department of Medical and Health Sciences, Linköping University, 581 83 Linköping, Sweden; Department of Clinical Physiology, Department of Medical and Health Sciences, Linköping University, 581 83 Linköping, Sweden

## Abstract

**Introduction:**

Myocardial dysfunction is a well-known complication in septic shock but its characteristics and frequency remains elusive. Here, we evaluate global longitudinal peak strain (GLPS) of the left ventricle as a diagnostic and prognostic tool in septic shock.

**Methods:**

Fifty adult patients with septic shock admitted to a general intensive care unit were included. Transthoracic echocardiography was performed on the first day, and repeated during and after ICU stay. Laboratory and clinical data and data on outcome were collected daily from admission and up to 7 days, shorter in cases of death or ICU discharge. The correlation of GLPS to left ventricular systolic and diastolic function parameters, cardiac biomarkers and clinical data were compared using Spearman’s correlation test and linear regression analysis, and the ability of GLPS to predict outcome was evaluated using a logistic regression model.

**Results:**

On the day of admission, there was a strong correlation and co-linearity of GLPS to left ventricular ejection fraction (LVEF), mitral annular motion velocity (é) and to amino-terminal pro-brain natriuretic peptide (NT-proBNP) (Spearman’s ρ -0.70, −0.53 and 0.54, and R^2^ 0.49, 0.20 and 0.24, respectively). In LVEF and NT-proBNP there was a significant improvement during the study period (analysis of variance (ANOVA) with repeated measures, p = 0.05 and p < 0.001, respectively), but not in GLPS, which remained unchanged over time (p = 0.10). GLPS did not correlate to the improvement in clinical characteristics over time, did not differ significantly between survivors and non-survivors (−17.4 (−20.5-(−13.7)) vs. -14.7 (−19.0 - (−10.6)), p = 0.11), and could not predict mortality.

**Conclusions:**

GLPS is frequently reduced in septic shock patients, alone or in combination with reduced LVEF and/or é. It correlates with LVEF, é and NT-proBNP, and remains affected over time. GLPS may provide further understanding on the character of myocardial dysfunction in septic shock.

## Introduction

Myocardial dysfunction in septic shock was first described as a condition of systolic depression despite normal or high cardiac output, demonstrating reversibility on remission [1,2]. Since the introduction of echocardiography in intensive care clinical practice, the character and incidence of myocardial dysfunction in septic shock has been studied further. Systolic as well as diastolic dysfunction, or a combination of the two, has been described, but the true characteristics of this particular septic organ failure remain unclear. Moreover, there is still lack of consensus on the definition and clinical spectrum of this entity. Numerous studies have investigated the prognostic importance of myocardial depression in septic shock and its impact on mortality, with conflicting results [3-6].

Echocardiographic measurement of systolic function by assessing left ventricular ejection fraction (LVEF) is dependent on volume and pressure load on the myocardium, and the systolic function may be overestimated in cases of severe septic vasodilatation [7]. The accuracy of echocardiographic measurements of diastolic dysfunction by Doppler measurements of blood or tissue velocity is dependent on the ultrasonic angle towards the tissue. Furthermore, mechanical ventilation, vasopressor use and vasoplegia, all of which are common in septic shock patients, pose challenges to a correct echocardiographic assessment [8]. In recent years, strain echocardiography, an echocardiographic method measuring global left-ventricular longitudinal myocardial deformation (global longitudinal peak strain, GLPS), has been introduced. This method has been claimed to be less pressure- and angle-dependent and in cardiology settings has been shown to be more sensitive in detecting cardiac dysfunction than conventional echocardiography [9]. In a paediatric population with septic shock, early myocardial dysfunction was identified with strain echocardiography [10], and in a recent experimental study in porcine septic shock, strain echocardiography revealed myocardial dysfunction before significant changes in LVEF were seen [11]. Furthermore, a recent clinical study in severe sepsis and septic shock patients has shown similar results with a higher prevalence of impaired GLPS than LVEF [12].

With the high mortality in septic shock, the recognition of complicating factors, in this case myocardial dysfunction, is paramount. However, the role of strain echocardiography in septic shock patients is not yet established. The aim of this study was therefore to evaluate strain echocardiography as a diagnostic and prognostic tool in septic shock by examining its relationship to other commonly used left ventricular function parameters, to cardiac biomarkers and clinical parameters, its change over time and its relation to outcome. We hypothesize that strain echocardiography can be used as a sensitive tool in the early recognition of septic cardiac dysfunction.

## Materials and methods

This study was approved by the Regional Ethical Review Board in Linköping, Sweden (Dnr 2012/233-31). When possible, informed consent was sought from patients at inclusion. Due to the observational nature of the study we were permitted to assume consent in patients who because of acute illness were unable to give informed consent. In these cases, informed consent was obtained as soon as possible after recovery.

Patients aged 18 years or older, admitted to the mixed non-cardiothoracic ICU of Linköping University Hospital, presenting with septic shock and with an expected ICU stay of 24 hours or longer, were screened for eligibility. In total, 50 patients were included from October 2012 to September 2014. Patients could be included only once. Patients who were not expected to survive longer than 24 hours according to the treating physician, patients in whom intensive care treatment was partly withheld from admission, and patients who due to language barriers or mental inability were not expected to be able to give consent even after recovery, were excluded. The study period was from ICU admission and up to 7 days, but shorter in cases of ICU discharge or death.

Septic shock was defined in the presence of the following international criteria [13]: (1) evidence or clinical suspicion of infection; (2) two or more of the following signs of systemic inflammatory response syndrome: (a) temperature >38 or <36°C, (b) pulse >90 bpm, (c) respiratory rate >20 breaths/minute or mechanical ventilation, and (d) white blood cells >12,000 μL^−1^ or <4,000 μL^−1^ or >10% bands; (3) at least one organ dysfunction; and (4) systolic BP <90 despite fluid therapy and requiring vasopressor therapy. All patients were treated according to international guidelines for the treatment of septic shock [13], and after initial resuscitation, at the discretion of the treating clinician. Standard echocardiographic and laboratory results were not concealed to treating physicians, but were not goals to which therapy was titrated. Patients were considered to have a history of cardiac disease if they had prior or current ischaemic heart disease, cardiac surgery, hypertension or cardiac failure.

Transthoracic echocardiography was performed as early as possible on the day of admission, and again after initial resuscitation, on day 3 or 4. In survivors, a follow-up echocardiogram was performed 8 to 30 days after inclusion. All echocardiograms were performed by an expert echocardiographer not involved in patient care (JE), using a Vivid E9 ultrasound scanner, acquiring two-dimensional apical two-chamber, four-chamber and long-axis views (2C, 4C and ALAX) with focus on the left ventricle (LV) and at a frame rate of >40 frames/s. Images were analysed offline using dedicated software (EchoPac version 112, GE Ultrasound, Horten, Norway), all by one observer (LDG). GLPS was calculated as the average speckle tracking strain from each of the 18 LV segments from the 2C, 4C and ALAX views (six segments per view, base-mid-apex, in three views). LV volumes and (LVEF were calculated using the modified biplanar Simpson method. E- and A-velocities and E-deceleration time were measured using pulsed wave (PW) Doppler in the mitral inflow at the tip of the valve. Diastolic tissue velocity of the base of the LV septum (é) was measured in the apical 4C view using PW tissue Doppler, and E/é ratios were calculated. All echocardiographic studies were recorded over three consecutive cardiac cycles, independently of breathing cycles, and averaged. Measurements were collected and averaged over 5 to 10 heartbeats in patients with non-sinus rhythm. To assess interobserver variability, all images were also independently analysed by an expert echocardiographer (JE). GLPS was considered decreased when > −15% [14]. Systolic dysfunction was defined as LVEF <50% [15], and diastolic dysfunction as E/é >15 and/or é <0.08 m/s [16].

Plasma concentrations of NT-proBNP and high-sensitivity Troponin T were analysed daily during the study period, the first sample being obtained as early as possible after ICU admission, using a Cobas® e 411 (Roche Diagnostics, Mannheim, Germany).

In patients where invasive continuous haemodynamic monitoring by means of thermodilutional technique was considered indicated by the treating physician, the commercially available PiCCO® system (Pulsion Medical, Munich, Germany) was used. The system was calibrated at least thrice daily, and cardiac index (CI) and systemic vascular resistance index (SVRI) from continuous measurements were collected when echocardiography was performed.

Clinical data on comorbidities, ventilator mode and settings, heart rate and rhythm, arterial blood pressure, fluid balance, vasopressor and inotropic infusion rates, ICU length of stay and outcome, were all collected prospectively. To assess severity of illness, the simplified acute physiology score 3 (SAPS 3) [17] was calculated on admission, and the sequential organ failure assessment (SOFA) score [18] was calculated daily.

### Statistical analysis

Data are presented as median (lower quartile to upper quartile) and number (percentage), as appropriate. For comparison between groups, the Mann-Whitney *U*-test and chi-square (χ^2^) test was used for continuous and dichotomous variables, respectively. The correlation between variables was explored using Spearman’s rank correlation test, and for temporal changes, repeated-measures analysis of variance (ANOVA) was applied. Univariate linear regression analysis was used to explore the explanatory value of variables, and a logistic regression model was calculated to determine the ability of variables to predict mortality. Interobserver variability of echocardiographic parameters was determined by the intraclass correlation coefficient. All probability values are two-tailed and significance was set at *P* <0.05. All statistical analyses were performed using IBM SPSS v22.0 (IBM Corp, Armonk, NY, USA) and STATA v11.1 (Stata Corp LP, College Station, TX, USA).

## Results

This study included 50 patients in septic shock, who all fulfilled the inclusion criteria of the study on admission. Table 1 summarizes the main clinical characteristics of included patients. The source of sepsis was pulmonary in 17 patients (34%), gastrointestinal in 11 patients (22%) and genitourinary in 11 patients (22%), and 7 patients (14%) were neutropenic, with haematological malignancies. Blood cultures were positive in 24 patients (48%), and all patients were treated with antibiotics chosen by an infectious disease specialist.

**Table 1:** **Baseline characteristics of studied patients on day 1**

**Demographics**	
Patients, n	50
Sex, male, n (%)	31 (64)
Age, years, median (IQR)	65 (58 to 74)
Weight, kg, median (IQR)	71 (65 to 82)
Cardiac comorbidities, n (%)	24 (48)
**Clinical data**	
SAPS 3, median (IQR)	73 (45 to 84)
SOFA day 1, median (IQR)	11 (9 to 12)
Mechanical ventilation, n (%)	39 (78)
Mechanical ventilation, hours, median (IQR)	67 (3 to 240)
Time in shock, hrs, median (IQR)	13 (8 to 22)
CRRT during ICU stay, n (%)	13 (26)
ICU length of stay, days, median (IQR)	5 (2 to 11)
**Mortality**	
Death in ICU, n (%)	13 (27)
Death within 30 days, n (%)	15 (30)
Death within 90 days, n (%)	17 (34)

Data are presented as medians (lower quartile to upper quartile) and number (n) of patients (%), as appropriate. SAPS, simplified acute physiology score; SOFA, sequential organ failure assessment; CRRT, continuous renal replacement therapy.

The first echocardiographic examination was performed as early as possible after ICU admission, and always on day 1. Two patients died before the echocardiogram was performed, in three patients image quality was inadequate, and in one patient, images were lost in the storage process. Thus, in 44 patients (88%), echocardiographic images could be analysed. On average, strain could be measured in 17 of the 18 LV segments. Of the 44 patients in whom echocardiography was performed after admission and where images were suitable for analysis, 37 patients (84%) were tracheally intubated and mechanically ventilated at the time of the examination. In survivors not yet discharged from the ICU, in total 26 patients, a second echocardiogram was performed after initial stabilisation (median 57 hours, IQR 48 to 82 hours after the first echocardiogram). In eight survivors who had not been transferred to referral hospitals, a follow-up echocardiogram was performed after discharge, 8 to 30 days after ICU admission and inclusion.

**Figure 1:**
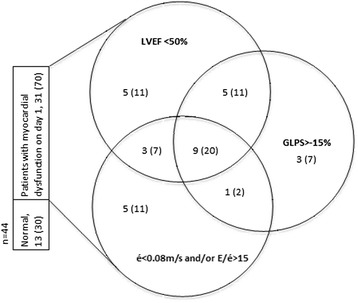
**Clinical spectrum and distribution of myocardial dysfunction on day 1 in studied patients.** Data are presented as number (percentage). LVEF, left ventricular ejection fraction; global longitudinal peak strain; é, early mitral tissue Doppler velocity; E/é, ratio of early mitral inflow (E) to é.

Overall 31 patients (70%) had LV dysfunction at the first examination. Decreased GLPS was seen in 18 patients (41%), whereas decreased LVEF was present in 22 patients (50%). Signs of diastolic dysfunction with decreased é velocity or increased E/é ratio were present in 18 patients (41%). As shown in Figure 1, there was marked overlap between groups, with 15 patients (34%) having decreased GLPS as well as decreased LVEF and/or diastolic dysfunction. Of those examined a second time, that is, alive but not yet discharged, there was LV dysfunction in 16 patients (62%), all of whom had decreased GLPS in combination with other echocardiographic signs of myocardial dysfunction.

**Figure 2:**
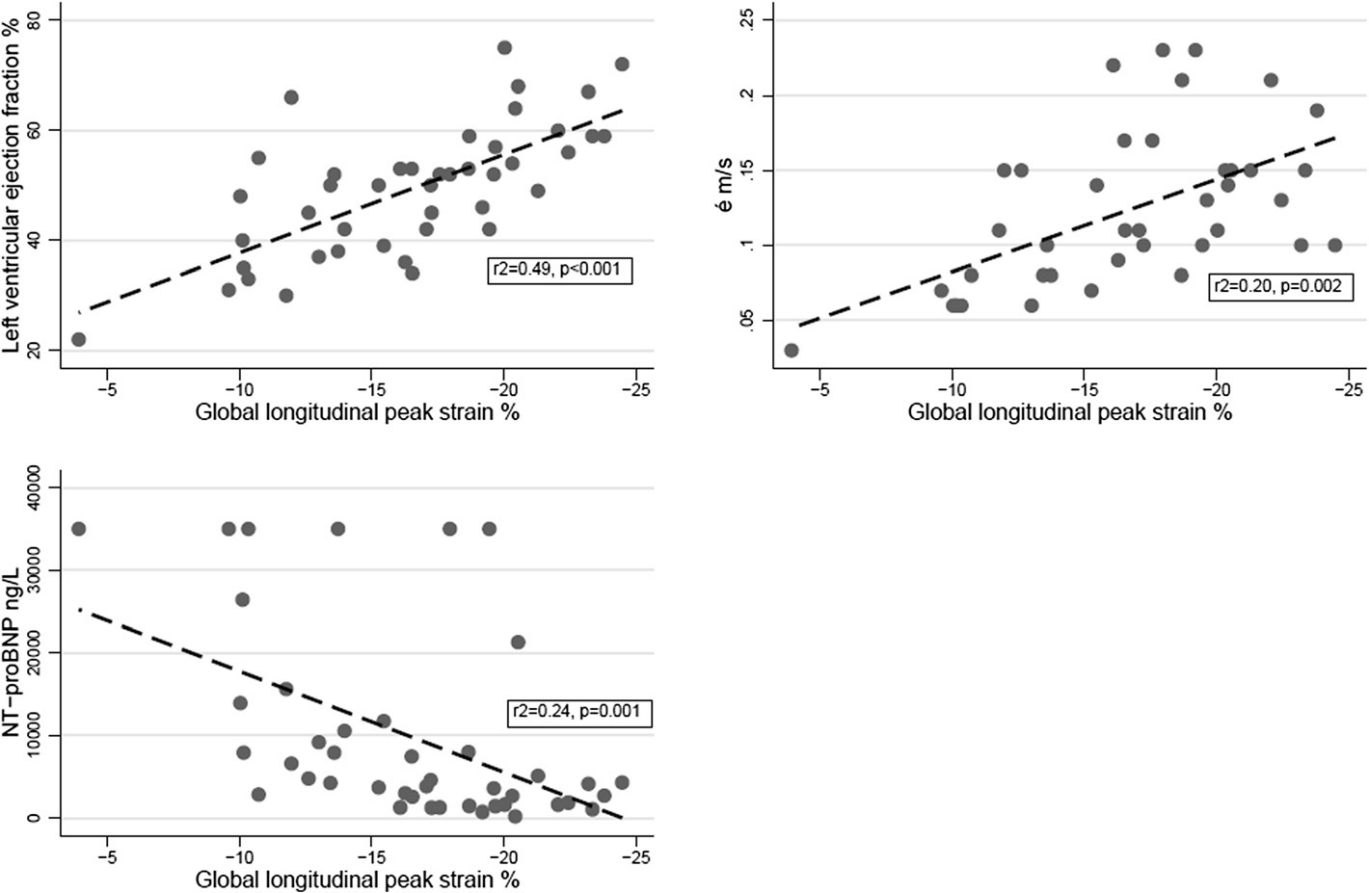
**Association of echocardiographic and laboratory markers of myocardial dysfunction.** The regression lines between global longitudinal peak strain and left ventricular ejection fraction, mitral annular motion velocity (é) and amino-terminal pro-brain natriuretic peptide (NT-proBNP) on day 1.

Table 2 shows the strong correlation of GLPS to LVEF, é, NT-proBNP and cardiac index on day 1. On the second echocardiogram, performed after initial stabilisation, and on the follow-up examination, the correlation was weaker. GLPS did not at any time correlate to SVRI or vasopressor dose, as measures of afterload, neither did it correlate to respiratory pressures, volume load or fluid balance, nor to SOFA score. Figure 2 shows the explanatory value of GLPS on echocardiographic parameters and NT-proBNP in a linear regression model. GLPS provided no explanatory value on clinical parameters.

**Table 2:** **Correlation of global longitudinal peak strain to left ventricular systolic and diastolic parameters, cardiac biomarkers and haemodynamics**

	**Global longitudinal peak strain**					
	**Echocardiography on day 1**		**Echocardiography on day 3 to 4**		**Follow-up echocardiography**	
**Echocardiographic parameters**	***r***	***P***	***r***	***P***	**r**	**p**
Left ventricular ejection fraction, %	−0.70	<0.001	−0.44	0.03	0.44	0.49
Early mitral tissue Doppler velocity, cm/s	−0.59	<0.001	−0.39	0.06	0.12	0.77
Ratio of early mitral inflow to early mitral tissue Doppler velocity	0.27	0.01	0.15	0.52	0.36	0.39
**Cardiac biomarkers and data from invasive monitoring**						
Amino-terminal pro-brain natriuretic peptide	0.54	<0.001	0.01	0.98		
High sensitivity troponin T	0.35	0.02	0.25	0.30		
Cardiac index at time of echo	−0.45	0.03	−0.54	0.25

**Figure 3:**
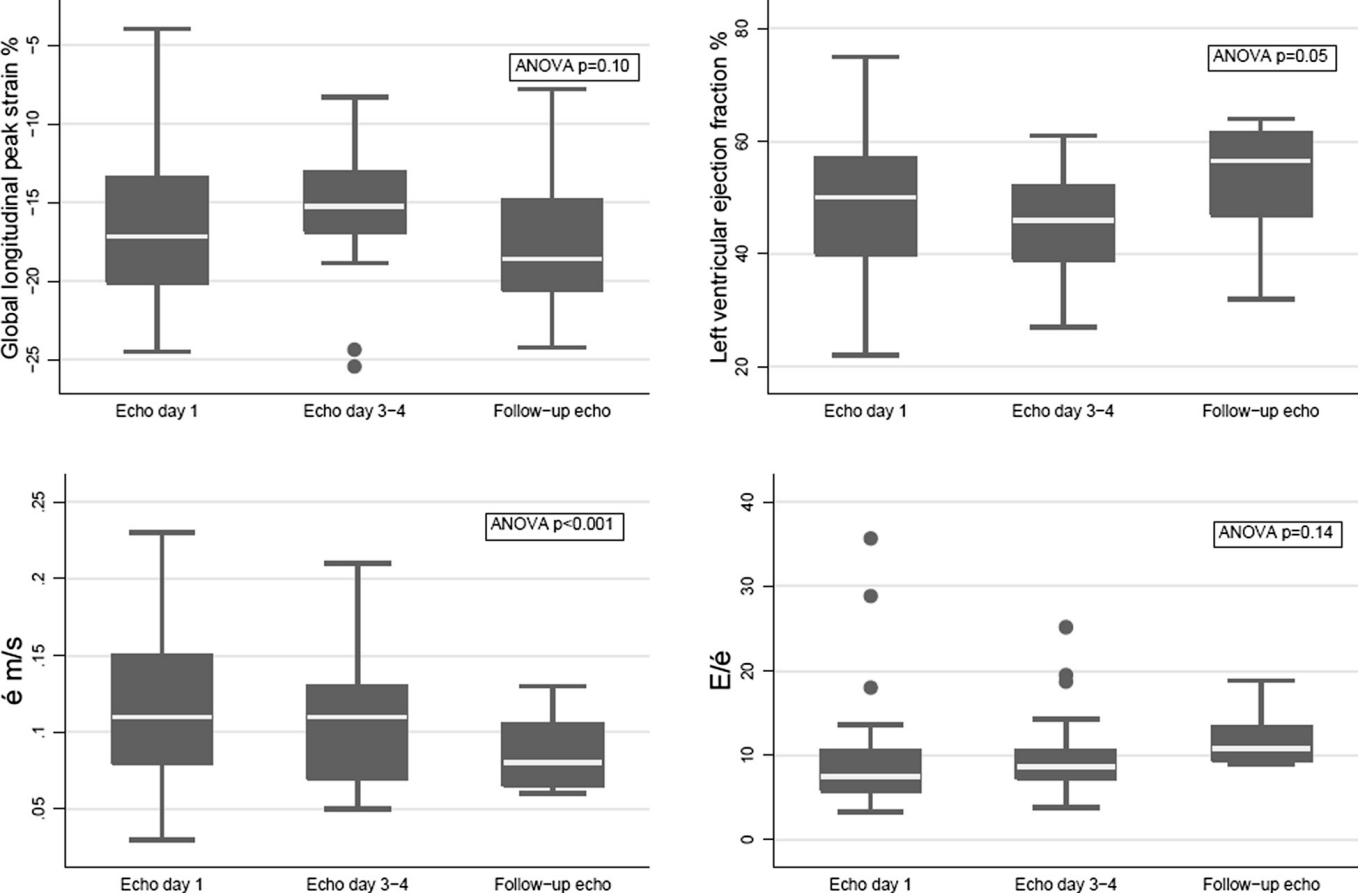
**Echocardiographic characteristics over time in studied patients.** The dispersion (minimum, 25th percentile, median, 75th percentile, maximum) and change over time of global longitudinal peak strain, left ventricular ejection fraction, mitral annular motion velocity (é) and early mitral inflow to mitral annular motion velocity ratio (E/é) at echocardiogram on day 1, after stabilization and on follow up, and their change over time. ANOVA, analysis of variance.

**Figure 4:**
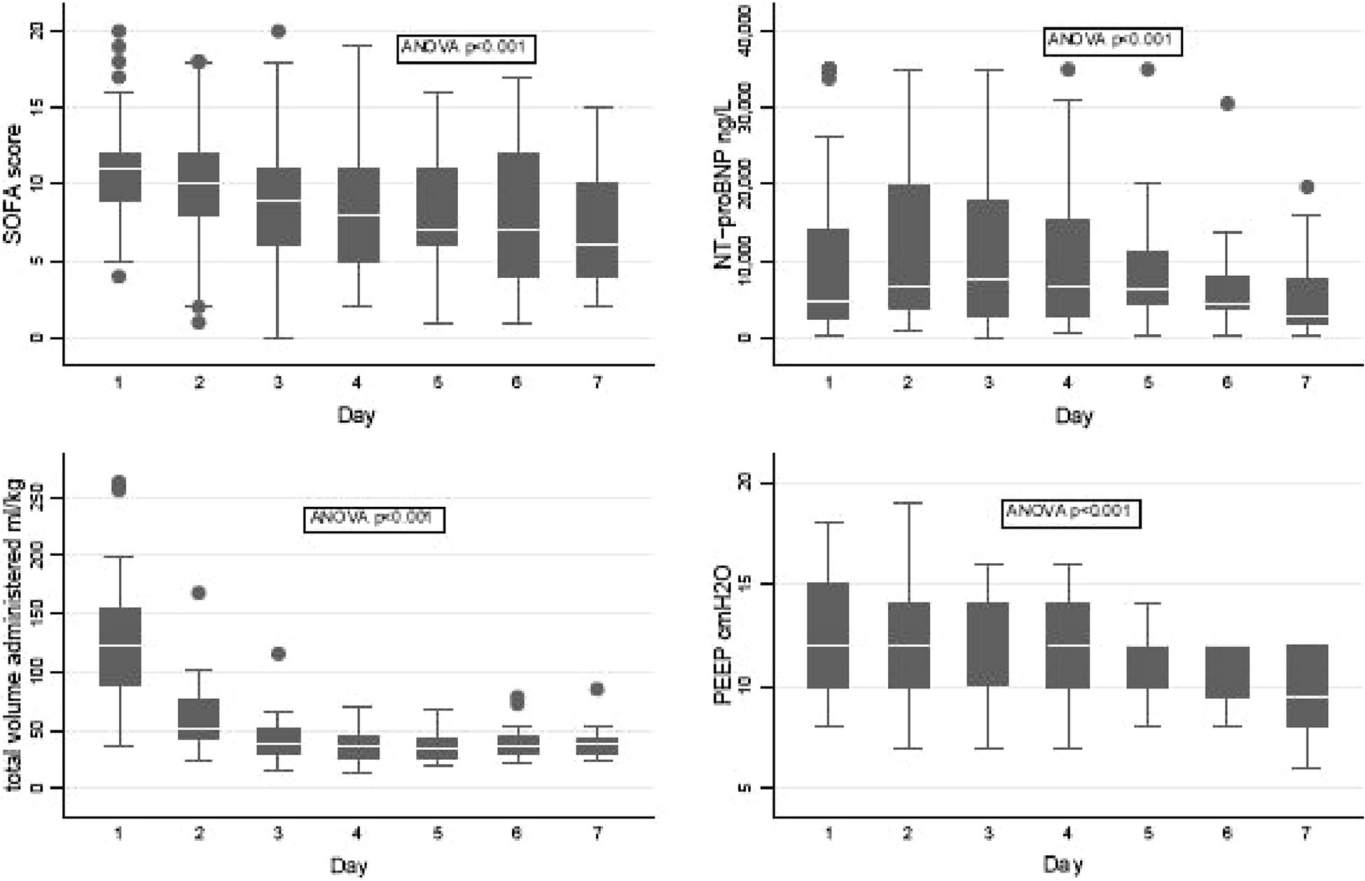
**Clinical characteristics over time in studied patients.** The dispersion (minimum, 25th percentile, median, 75th percentile, maximum) and change over time of sequential organ failure assessment (SOFA) score, amino-terminal pro-brain natriuretic peptide (NT-proBNP), the total volume of fluids administered and the peak positive end-expiratory pressure (PEEP) in studied patients on each day during the study period. ANOVA, analysis of variance.

Figure 3 shows the change in GLPS, LVEF, é and E/é over time during the study period. All echocardiographic parameters showed a large dispersion from day 1 to follow up. LVEF and é showed significant change over time, whereas GLPS and E/é did not. The day-to-day course of laboratory and clinical parameters, with SOFA score, NT-proBNP, volume load administered and positive end-expiratory pressure PEEP all showing significant changes over time are presented in Figure 4.

Of the 50 patients included, 13 patients (26%) died in the ICU. Another two patients died within 30 days, and 17 patients (34%) had died within 90 days. Table 3 summarizes the main clinical, laboratory and echocardiographic characteristics in survivors and non-survivors at 30 and 90 days, with a significant difference between groups in SAPS 3, age, ICU length of stay and NT-proBNP on admission. GLPS was the only echocardiographic parameter with a tendency to differ between survivors and non-survivors. A logistic regression model showed that GLPS >−15 gave a hazards ratio for death at 90 days of 2.5, but the result was not statistically significant (95% CI 0.66, 9.46), and no predictive value was gained by using a multivariable model with LVEF, é and NT-proBNP in addition to global strain. The intraclass correlation coefficient analysing interobserver variability of GLPS was 0.92 (*P* <0.001).

**Table 3:** **Patient characteristics on ICU admission (day 1) in comparison between survivors and non-survivors**

	**30 Days**		***P***	**90 Days**		***P***
	**Survivors, n = 35**	**Non-survivors, n = 15**		**Survivors, n = 33**	**Non-survivors, n = 17**	
**Demographics**						
Male sex, n (%)	24 (69)	7 (47)	0.12	18 (55)	9 (53)	0.54
Age, years, median (IQR)	65 (56 to 71)	75 (65 to 79)	0.01	64 (54 to 71)	72 (65 to 79)	0.02
Weight, kg, median (IQR)	74 (65 to 85)	68 (64 to 80)	0.42	72 (63 to 84)	70 (64 to 81)	0.86
Cardiac comorbidities, n (%)	15 (43)	9 (60)	0.23	14 (42)	10 (59)	0.17
**Clinical data and cardiac biomarkers**						
Saps 3, median (IQR)	70 (59 to 76)	81 (70 to 88)	0.03	68 (59 to 76)	81 (70 to 88)	0.01
ICU length of stay, days, median (IQR)	115 (56 to 272)	93 (23 to 142)	0.13	168 (81 to 290)	78 (23 to 143)	0.02
Mechanical ventiltion, hours, median (IQR)	70 (0 to 240)	45 (9 to 135)	0.77	154 (19 to 245)	40 (2 to 118)	0.17
Sequential organ failure assessment score	10 (9 to 12)	12 (9 to 14)	0.62	10 (9 to 12)	11 (9 to 14)	0.61
PEEP, cmH2O	14 (10 to 16)	12 (10 to 15)	0.73	12 (10 to 15)	12 (10 to 15)	0.91
Pmax, cmH2O	28 (22 to 31)	32 (26 to 34)	0.18	28 (21 to 31)	32 (26 to 34)	0.21
Norepinephrine, ug/kg/min	0.09 (0.05 to 0.14)	0.11 (0.05 to 0.26)	0.50	0.09 (0.06 to 0.16)	0.08 (0.04 to 0.22)	0.95
Dobutamine, ug/kg/min	5.5 (3.1 to 8.2)	5.4 (4.7 to 11.3)	0.46	5.1 (3.1 to 8.8)	5.6 (4.8 to 10.4)	0.34
Fluids administered, ml/kg	127 (100 to 154)	89 (60 to 200)	0.34	131 (100 to 154)	90 (62 to 118)	0.15
Fluid balance, ml/kg	70 (33 to 95)	21 to 151)	0.99	71 (31 to 104)	59 (23 to 132)	0.96
Cardiac index, l/min/ m^2^	4.0 (3.3 to 5.0)	5.0 (4.6 to 5.4)	0.14	4.0 (3.2 to 5.1)	4.9 (4.3 to 5.4)	0.16
SVRI dyn × sec × cm^−5^/m^2^	1310 (1066 to 1582)	800 (600 to 852)	0.001	1,310 (1,062 to 1,585)	801 (600 to 1045)	0.002
NT-proBNP, ng/L	4230 (1410 to 10400)	7940 (2780 to 26400)	0.15	4,070 (1,400 to 8,510)	10,500 (2,860 to 30,700)	0.03
High sensitivity troponin T, ng/L	40 (24 to 119)	121 (39 to 530)	0.09	40 (26 to 236)	115 (42 to 348)	0.08
**Echocardiography**						
Global longitudinal peak strain, %	−17.2 (−20.0 to (−13.0))	−15.0 (−19.7 to (−11.0))	0.34	−17.4 (−20.5 to −13.6)	−14.7 (−19.0 to −10.6)	0.11
Left ventricular ejection fraction, %	50 (42 to 57)	53 (37 to 59)	0.99	50 (44 to 58)	47 (36 to 56)	0.47
E/é	7.1 (5.9 to 11.0)	7.7 (5.4 to 10.7)	0.62	7.4 (6.0 to 11.7)	7.4 (5.7 to 9.0)	0.60
é, m/s	0.11 (0.08 to 0.18)	0.12 (0.83 to 0.16)	0.99	0.11 (0.08 to 0.16)	0.11 (0.08 to 0.16)	0.95
E, m/s	0.97 (0.79 to 1.07)	0.95 (0.61 to 1.07)	0.53	0.98 (0.79 to 1.08)	0.95 (0.60 to 1.11)	0.46
A, m/s	0.73 (0.57 to 0.92)	0.99 (0.845 to 1.20)	0.31	0.73 (0.59 to 0.89)	0.81 (0.47 to 1.48)	0.34
E/A	1.2 (1.0 to 1.7)	1.0 (0.8 to 1.2)	0.10	1.2 (1.0 to 1.7)	1.1 (0.9 to 1.2)	0.60
DT, ms	157 (140 to 197)	171 (105 to 200)	0.97	156 (140 to 199)	172 (126 to 204)	0.93

Data are presented as median (IQR) or number (%), as appropriate. SAPS, simplified acute physiology score; Pmax, peak inspiratory pressure; PEEP, positive end-expiratory pressure; SVRI, systemic vascular resistance index; NT-proBNP, amino-terminal pro-brain natriuretic peptide; E: early mitral inflow; é: early mitral tissue Doppler velocity; A: late mitral inflow; DT: early mitral inflow deceleration time.

## Discussion

In this study we evaluate strain echocardiography for detecting LV dysfunction in patients with septic shock. The study demonstrates that GLPS is frequently impaired in septic shock patients irrespective of survival, and that GLPS correlates with other echocardiographic and laboratory signs of cardiac dysfunction. Furthermore, the study suggests that GLPS remains unchanged over time, in spite of normalizing systolic function parameters, cardiac biomarkers, and clinical recovery.

The landmark study first describing cardiac dysfunction in septic shock showed systolic impairment in 10 of 20 patients during the first 48 hours [1]. Later studies have reported similar results with a reduced global systolic function in 30 to 60% of septic shock patients [5,19], and reversibility in those who survived [8,20,21]. The definition of systolic dysfunction has varied among previous studies (LVEF <50 to 55%), which may in part explain the varying incidence. The results of this study are in line with these previous results, with reduced LVEF in 50% of patients and improvement in survivors. In recent years, especially since the introduction of tissue Doppler in intensive care practice, diastolic dysfunction in septic shock has gained interest. Several studies have shown decreased é and increased E/é in this setting, with a prevalence of 20 to 57% [3,4,21-23]. Differences in study size and characteristics in previous cardiac disease and clinical circumstances may have contributed to these differences. When using the same definition of diastolic dysfunction as in these studies (E/é >15 or é <0.08 m/s) we found a prevalence of 50%. In contrast to previous longitudinal studies, no improvement over time was seen [3,4,21,22].

The impact of myocardial dysfunction on mortality in septic shock is still unclear. When first described, LVEF was paradoxically reduced in survivors compared with non-survivors [1]. Later studies have confirmed this finding [6], whereas in other studies, and in ours, no difference in LVEF between survivors and non-survivors has been detected [5,23]. This may be related to the small sample size (n = 21 to 61) or to the large prevalence of pre-existing cardiac disease (43 to 59%) in these studies as well as in ours, but the finding has been confirmed by other, larger studies (n = 106 to 262) [3,4]. In contrast, a substantial impact of diastolic dysfunction on mortality in septic shock has been shown [23]. Larger studies have confirmed this finding, but have included patients with severe sepsis alongside those with septic shock, which complicates direct comparison with our study [3,4], where we saw no difference in systolic or diastolic parameters between survivors and non-survivors. GLPS was the only echocardiographic parameter that had a tendency to differ between survivors and non-survivors, but neither of the parameters could be used in a prognostic model. However, this is in line with two previous studies evaluating GLPS in sepsis [10,12].

In order to further characterize the nature of cardiac dysfunction in septic shock, other echocardiographic techniques have been used to investigate the systolic and diastolic function in sepsis. Tissue Doppler measures of the peak systolic velocity at the mitral annulus (Sa) in septic shock have shown a linear association with LVEF [5,24], and the association of Sa to mortality has been stronger than that of LVEF. Mitral annular plane systolic excursion (MAPSE) has been shown to correlate to LVEF as well as to E/é and é [25], and therefore is assumed to reflect systolic as well as diastolic function. Strain echocardiography, used in this study, describes tissue deformation, and GLPS is viewed as a measure of systolic function representing contractility. However, we demonstrated here the association of GLPS not only to LVEF but also to é, E/é and to NT-proBNP and cardiac index, and also a marked overlap of, or interaction between, systolic and diastolic dysfunction. Thus, while GLPS seems to reflect the systolic dysfunction, systolic dysfunction commonly coincides with diastolic dysfunction in patients with septic shock.

In a recent study on severe sepsis and septic shock patients, there was a higher prevalence of impaired GLPS than of reduced LVEF [12]. In contrast, we found reduced LVEF to be more frequent than reduced GLPS, although direct comparison between the two studies is complicated by differences in inclusion criteria, with all our patients being in septic shock. Also, our findings suggest that GLPS is relatively independent of SVRI, vasopressor and inotropes, volume loading and extrinsic ventilator pressure, all of which most certainly affect cardiac mechanics [6]. These findings correlate to those of a recent experimental study, where GLPS decreased in correlation with LVEF, but earlier in the course of septic shock, and was impaired in spite of a decreased SVRI [11]. GLPS has indeed been claimed to be relatively independent of pre-load and after-load, in addition to being less angle-dependent than tissue Doppler ultrasound [9]. Furthermore, we saw a significant improvement over time in systolic parameters as well as in clinical conditions over time, but no change in GLPS. This might indicate that a decreased GLPS could represent more subtle changes in the myocardium in septic shock that persist even after clinical recovery. In cardiological studies, GLPS has been sensitive in detecting myocardial disease such as hypertrophic cardiomyopathy [26], cardiac amyloidosis [27] and chemotherapy-associated cardiac dysfunction, even in asymptomatic patients [28]. Further studies are needed to investigate the importance, the time course and the long-term nature of decreased GLPS in septic shock patients.

### Limitations

This is a single-centre study, and local management strategies may have influenced patient selection as well as treatment and outcome. Our hospital is a tertiary care centre to which some patients are referred from other hospitals, where sepsis treatment may have started before admission. In addition, patient selection may have been biased by the strict exclusion criteria of the study, with the most severely ill patients not being included.

Second, the clinical spectrum of septic shock is wide and highly dynamic, and although all patients were included during the first 24 hours after ICU admission, time from initial presentation of disease to echocardiogram and other measurements varies. Also, the study would have been strengthened if echocardiography had been performed daily during the study period. Furthermore, treating physicians were not blinded to measurements or results, which may have influenced the treatment of patients. Still, our intention was not to study differences in treatment, and the clinician’s treatment decisions should always be based on all available data. Finally, the group of studied patients is small, and cannot be expected to provide definite conclusions about outcome.

The strength of the study lies in the parallel use of echocardiographic measurements, biomarkers and clinical data analysed repeatedly throughout the most unstable period. Further strength lies in survivors being followed up to investigate the normalization over time of myocardial dysfunction in sepsis, as described by others. Moreover, as strain echocardiography represents a computer-generated measurement, it is potentially more objective and possibly less user-dependent, than other echocardiographic measurements.

## Conclusion

We conclude that GLPS is frequently reduced in intensive care patients with septic shock, survivors as well as non-survivors. The impairment of GLPS correlates to that of systolic as well as diastolic LV function parameters and cardiac biomarkers, but remains unchanged over time, even after clinical recovery. The study provides additional insights into the pathophysiology of septic myocardial depression, and together with further studies the results may question the accepted view on septic cardiac depression as a septic organ failure that resolves with clinical recovery.

## Key messages

- Strain echocardiography can be used to show LV dysfunction in septic shock
- GLPS correlates with other echocardiographic and laboratory markers of myocardial dysfunction
- GLPS is frequently impaired in survivors as well as non-survivors of septic shock
- GLPS remains unchanged in contrast to normalizing systolic function parameters and biochemical markers

## Abbreviations

2C: two-chamber view
4C: four-chamber view
A: late mitral inflow
ALAX: apical long-axis view
ANOVA: analysis of variance
ARF: acute renal failure
CI: cardiac index
CRP: C-reactive protein
DT: early mitral inflow deceleration time
E: early mitral inflow
é: early mitral tissue Doppler velocity
E/A: E to A ratio
E/é: E to é ratio
GLPS: global longitudinal peak strain
hsTnT: high sensitive troponin T
LV: left ventricle/ventricular
LVEF: left ventricular ejection fraction
NT-proBNP: amino-terminal pro-brain natriuretic peptide
PCT: procalcitonin
PEEP: positive end-expiratory pressure
PiCCO: pulse contour continuous cardiac output
Pmax: peak inspiratory pressure
PW: pulsed wave Doppler
Sa: peak systolic velocity measured at the mitral annulus
SAPS3: simplified acute physiology score 3
SOFA: sequential organ failure assessment
SVRI: systemic vascular resistance index
